# American Medical Association

**Published:** 1851-09

**Authors:** 


ARTICLE II.

### AMERICAN MEDICAL ASSOCIATION.

*Report of the Committee on Practical Medicine.* AUSTIN FLINT, M. D., Prof., &c., Chairman.

In our last we promised to give a more extended notice of this document. We now proceed to redeem that promise. As has already been observed, the report is systematic and well written—taken altogether we think it decidedly the best report upon the subject that has yet been submitted to the Association.

It commences by an account of the important improvements in the treatment of individual diseases. In his classification he follows the nosological arrangement of Dr. Goode, beginning with the diseases that affect the Digestive Organs.

Treatment of Dysintery by injections of the Nitrate of Silver, is well advocated by various authors, in the report. Among them are Dr. Lewis Slusser, of Ohio; Dr. Thweatt, of Va.; Dr. McGugen, of Iowa; Dr. Matthews, of Ind.; and Dr. N. A. J. Riddle, of Alabama.

Its treatment by the free use of Morphine, is advocated by Dr. Tullis of Troy, and Dr. Murphy of Cincinnati, Ohio; and by Dr. J. D. Trask, of N. Y.

Mr. Mussey, of Cincinnati, O., has found the granulated Tin an efficient remedy in Ternia.

Dr. Dawson, of the Commercial Hospital, Cincinnati, has reported his experience of the institution in the use of 'Wood Naptha in Diarrhœa, which shows very favorable success.

Doctor Jewett, of Maine, reports a case of Icterius treated by Sulph. Manganese with prompt success. But there is no disease (or symptom) more liable to deceive, as it often very rapidly recovers by the unaided efforts of nature.

First among the articles upon diseases of the respiratory Apsaratus noticed, are those of Prof. Ware, of Boston, on Croup, which are regarded as important.

The use of Nitric Acid in Asthma, has been recommended by Dr. Hopkins, of Georgia. He gives from five to ten drops three times a day in syrup or water.

The subject of withdrawing fluids from the chest by means of an Exploring Trochar and Canula attached to an exhausting pump, as practiced by Dr. Bowditch, of Boston; the use of Cod Liver Oil and the inhalation of Medicated powder, as recommended by Dr. Cornell, of Boston, close this part of the subject.

In reference to the management of diseases affecting the Nervous System, the report notices the accounts given by Dr. Guerard of S. C., and Dr. Satterlee of Wis., of the treatment of Hydrocephalus by the exhibition of Iodide of Potassium, in which its virtues are highly recommended.

Dr. Glenn, of S. C., and Dr. Gantt, of Md., have both treated Tetanus successfully with the Cannabis Indica.

Dr. Lebby, of S. C., has treated four cases of the same formidable malady successfully, by inducing Mercurial Salivation.

Dr. Smith, of N. J., has treated a case of Tranmatic Tetanus, in which the beneficial effects of Chloroform were manifested.

Dr. Hook, of S. C., has treated a case of Sciatic Neuralgia by the application of Chloroform to the part by a saturated cloth, in which the relief was prompt and permanent. Dr. Holmes relieved a similar case by administering the same remedy internally.

The only notice in reference to the treatment of diseases of the Genito-Urinary organs, is the of treatment of Spermatorrhœa by Strychnine and Veratria Ointment, recommended by Dr. Campbell, of Georgia.

In reference to Typhoid fever the report is fuller, and quotes various authors, giving diversified modes of treatment.

We are well satisfied with the success that has attended the treatment of Typhoid fever with well salted animal broths, with but little medicine, in the Illinois General Hospital. Our limits will not allow a full synopsis of this part of the report, although it is very interesting.

Different authors bear testimony to the success of inunction in Scarlatina, as practiced by Dr. Schneeman. We have in several cases seen its good effects.

Notice is made of the treatment of Rubeola by the same means as proposed in an article we published last November.

Dr. McGirr's article on inoculation in Rubeola, receives an extended notice.

Dr. Bell, of Louisville, Ky., prefers as an *ectrotic* in Variola, a strong murcurial ointment, with two drachms of starch to the ounce.

Dr. Brinkerhoff, of Pa., proposed the application of Collodion to prevent pitting in this disease.

The abortive treatment of Intermittent fever by the free use of Quinine, is discussed by Dr. Bell. So general is the adoption of this course of treatment in our country now, that we deem it unimportant to give the arguments of the author. However, as the report is for the whole country, the space devoted to the use of Quinine is well used, and we hope it may do good where the profession is behind the times on this point. Notice is made of Prof. Brainard's treatment of Intermittents with Strychnine.

Dr. Upshur, of Virginia, speaks favorably of the use of Croton Oil, as a cathartic, in Rheumatism ; next to it in point of importance he recommends the use of Quinine.

The tendency to employ leading articles of the materia medica indiscriminately is so strong, that we have been fearful that Quinine is destined to almost as universal an employment as Calomel has had. Though we regard it as a much safer remedy to be thus used, we hope the profession, now that it has before it the lesson of the calomel mania, will be careful not to carry the use of quinine, or any other remedy, to the extent of giving it universal applicability to the cure of disease.

Dr. Norwood, of S. C., has proposed the use of *Veratram Viride*, or American Hellebore, for diminishing the force of the circulation in affections presenting this indication.

Prof. L. A. Dugas, of Ga., has called attention to the effects of large dilutions of certain remedies, such as Iodine, Arsenical preparations, Corrosive Sublimate, Calomel, &c. He thinks many advantages may be gained by dilution, a principle which the author of the report very justly regards as too often overlooked.

After calling attention to the remarks of Prof. Warren, on the use of cold Water as a Therapeutical agent, and some general observations upon sanitary measures, this part of the report is drawn to a close.

That part of the report treating of the progress of Epidemics, is full and quite catholic in its spirit, noticing all the accounts of Epidemics that had come to the knowledge of the Committee, but our space will not allow us to extend this notice further. We have noticed in preference the improvements in treatment, as both more interesting and more useful to our readers.

